# COVID19 vaccination in adults with sickle cell disease is not associated with increases in rates of pain crisis

**DOI:** 10.1080/16078454.2022.2085072

**Published:** 2022-12

**Authors:** Elana Friedman, Caterina Minniti, Sean Campbell, Susanna Curtis

**Affiliations:** a Albert Einstein College of Medicine, Bronx, NY, USA; b Montefiore Medical Center, Bronx, NY, USA

**Keywords:** Sickle cell disease, COVID-19, vaccination

## Abstract

People with sickle cell disease (SCD) are more vulnerable to hospitalization, pneumonia, and pain following COVID-19 infection. However, given the association between the inflammatory response and vaso-occlusive crises in SCD and a case report of vaso-occlusive crises following administration of the ChAdOx1 nCov-195-7/AstraZeneca vaccine, there is concern that the administration of COVID-19 vaccines in people with SCD might provoke a vaso-occlusive crisis. To address this critical gap in knowledge, we sought to examine acute care usage for vaso-occlusive crisis and frequency and severity of side effects following COVID-19 vaccination among patients at the Montefiore Sickle Cell Center for Adults. As part of regular care, patients were asked if they had received COVID-19 vaccination and any side effects were noted. Electronic medical records were reviewed for the type of vaccine, dates received, episodes of vaso-occlusive crises within seven days of a dose, and side effects noted. The risk of average hospital utilization per week in 2019 was calculated as a baseline. We found that fewer than 1 in 10 patients presented to the hospital within seven days of vaccination and that the risk of hospital utilization was similar to the average risk in a week in 2019. Of patients who reported side effects, one reported a possible case of sensorineural hearing loss otherwise no other rare side effects, including thrombosis or death, were reported.

## To the editor

Sickle cell disease (SCD) is one of the most common and severe monogenic disorders. In prior studies, people with SCD disease who became infected with COVID-19 had higher rates of hospitalization, pneumonia, and pain than the general population [1]. Based on this, the American Society of Hematology and the Sickle Cell Disease Association of America have recommended that people with SCD receive COVID-19 vaccination and booster and the CDC lists people with SCD as a population vulnerable to severe COVID [2]. However, some studies have noted increased vaccine hesitancy among people with SCD and in our clinic patients expressed concerns about the safety profile of the COVID-19 vaccines in people with SCD [3,4]. Further, given the known association of inflammation and sickle cell crisis, there is concern that an immune reaction to vaccination might provoke a vaso-occlusive crisis or a significant increase in vaccination’s known adverse effects [5]. A recent publication reported three patients with SCD who developed vaso-occlusive crises within six days following receipt of the ChAdOx1 nCov-195-7/AstraZeneca vaccine [6] which may raise concern regarding other COVID-19 vaccines. To date, no clinical trials have reported on the safety or efficacy of the COVID-19 vaccines administered in the USA; Pfizer mRNA, Moderna mRNA, or Jansen adenovirus, in people with SCD. To address this critical gap in knowledge, we sought to examine acute care utilization for pain crises and frequency and severity of side effects after receiving any COVID-19 vaccination approved in the USA among patients with SCD at our center.

Approval for this study was obtained from the Albert Einstein Institutional Review Board. All Patients with SCD who were seen as outpatients at the Montefiore Sickle Cell Center for Adults from 5/1/21 to 7/7/21 were included. As part of regular care, patients were asked if they had received the COVID-19 vaccination and any side effects were noted. Electronic medical records (EMR) were manually reviewed for data extraction about the type of vaccine (Pfizer mRNA vaccine (Pfizer Inc. New York, NY, USA), Moderna mRNA vaccine (Moderna Inc. Cambridge, MA, USA), or Janssen adenovirus vaccine (Janssen Inc. Titusville, NJ, USA), the dates received, episodes of pain crisis within seven days of a dose, and patient reported side effects were noted. Episodes of pain crises were defined as an episode of hospital utilization (Emergency Department (ED) or hospital admission) during which IV opioid analgesia was required. Clinical variables including age, sex, body mass index (BMI), race, genotype, medications, and history of SCD comorbidities were also noted.

Patients were classified as vaccinated if they had reported receiving at least one dose of Pfizer, Moderna, or Janssen and/or if vaccination was received at our medical center and documented in the EMR. If only one date of vaccination was present in the EMR and a vaccine card was not available, but the patient endorsed receiving two doses, the date for the second dose was based on the recommended vaccination schedule. Hospital utilization in 2019 was examined as a control. The risk of hospital utilization for any week in 2019 was calculated for each individual by dividing total hospital utilization in 2019 by 52. Rates of hospital utilization between first and second doses and between all doses and 2019 were calculated using paired student’s T test. We also assessed whether utilization of hydroxyurea – a disease-modifying therapy – had any effect on the presence of side effects or hospital utilization for pain crises after the first or second dose using a chi [2] method of analysis.

Of 123 patients with SCD in the sample, 87 (70.7%) were vaccinated and 36 (29.3%) were unvaccinated. Of the 71 vaccinated patients for whom type of vaccine was reported, 46 (64.8%) received the Pfizer, 22 (31.0%) received the Moderna, and 3 (4.2%) received the Janssen. Vaccinated patients were a median of 43 years old, 58.6% female, and 77.2% with SS/SB0 genotype (Table 1). Of 65 patients with a known date of vaccination, 2 (3.13%, 95%CI: 1.26–8.51%) presented to the hospital with a sickle cell crisis within one week of the first dose (1 ED visit and 1 admission for uncomplicated pain crisis) and 4 (6.67%, 95%CI: 0.17–13.2%) presented to the hospital with a sickle cell crisis within one week of the second dose (1 ED visit, 2 admissions for uncomplicated pain crisis, 1 admission for pain crisis with acute chest which resolved with 1 unit red blood cell transfusion). In 2019, the mean risk of hospital utilization (both ED and admission) among the same cohort of patients in a week was 6.12% (95% CI 4.01–8.24%). The differences in risk of presentation to the hospital between doses were not significant (*p* = 0.36). For the 65 patients examined, the rate of hospitalization utilization within a week after the first or second COVID-19 vaccination was not different from the rate of hospital utilization in an average week in 2019 (1st vaccine *p* value = 0.43, 2nd vaccine *p* value = 0.87). Of 26 patients for whom documentation of other side effects was available, 15/26 (57.7%) reported one or more side effects including injection site soreness (2), chills (3), fatigue (3), body aches (5), headaches (1), gastrointestinal symptoms (2), and one patient who received Pfizer reported a possible case of sudden sensorineural hearing loss (SNHL) [7]. This patient was not seen nor treated for the SNHL at our center and we were unable to obtain the medical records from the event. Patient utilization of hydroxyurea had no significant effect on the presence of side effects post-vaccination (*p* = 0.457), likelihood of hospital utilization after the first dose (*p* = 0.711), or after the second dose (*p* = 0.570).

In conclusion, fewer than 1 in 10 patients with SCD who received COVID-19 vaccination presented to the hospital within 7 days of vaccination and the risk of presenting to the hospital after vaccination was similar to the average risk of presenting to the hospital in any week of 2019. Of 87 patients with SCD who received a COVID-19 vaccination, one developed possible sensorineural idiopathic hearing loss which resolved (this has been reported after COVID-19 vaccination), however no other rare side effects including thrombosis or death were reported. In addition, the presence of a disease-modifying therapy (hydroxyurea) did not have an effect on the presence of side effects or hospital utilization after either the first or second dose. Our study is limited in that it is a retrospective single center study. Larger prospective studies are needed to examine the incidence of rare side effects and the efficacy of vaccines in people with SCD. Given the nature of this study, it is not clear how many of these episodes may have been provoked by vaccination as opposed to other factors. Even an ‘uncomplicated pain crisis’ can be traumatic even for a person living with SCD. Moreover, our data represent vaso-occlusive crises that resulted in hospital utilization and may underrepresent episodes managed at home, especially during a global pandemic that left many patients reluctant to seek medical attention. However, considering the known increase in both vaso-occlusive crisis and acute chest provoked by COVID-19 infection in people with SCD, we will continue to encourage patients at our center to receive vaccinations. Anecdotally, sharing these data with patients in our clinic has helped to reassure them when discussing whether to receive a COVID-19 vaccination.

## Figures and Tables

**Table 1.** Clinical characteristics for vaccinated and unvaccinated patients.

Age, median (IQR)	43 (30/52)
Female (%)	58.6%
SS Genotype (%)	74.0%
African American (%)	80.5%
BMI, median (IQR)	23.6 (21.2/26.6)
Hydroxyurea (%)	63.2%
Voxeletor (%)	16.1%
L-glutamine (%)	8.0%
Stroke (%)	17.2%
Thromboembolism (%)	8.0%
Acute chest (%)	54.0%
Avascular necrosis (%)	41.4%
Ulcers (%)	27.6%
Priapism (%)	30.6%
